# High-Performance Thrombin Aptamers with a Peptide-Extended Recognition Interface

**DOI:** 10.1021/acsomega.6c05416

**Published:** 2026-07-07

**Authors:** Irina V. Varizhuk, Natalia A. Kolganova, Olga B. Gordeeva, Andrey A. Stomakhin, Diana A. Talipova, Sergei A. Surzhikov, Edward N. Timofeev

**Affiliations:** † Engelhardt Institute of Molecular Biology, Russian Academy of Sciences, 119991 Moscow, Russia; ‡ Petrovsky National Research Center of Surgery, 119991 Moscow, Russia; § Pirogov Russian National Research Medical University, 117997 Moscow, Russia; ∥ Sechenov First Moscow State Medical University, 119991 Moscow, Russia

## Abstract

Improving the affinity and inhibitory characteristics of aptamers is important in the context of their applications in therapy and diagnostics. The use of peptide–aptamer conjugates with an extended aptamer–protein interface is an efficient strategy toward this goal. Here, we report GLE peptide conjugates of the bimodular duplex–quadruplex thrombin aptamers Re31 and NU172. Biophysical studies of the aptamer conjugates revealed that the presence of the tripeptide subunit does not have a significant effect on the thermodynamic stability or structure of the aptamers. In contrast, the affinity and anticoagulant activity of the conjugates were significantly improved. The NU172–GLE conjugate appeared to be the most effective inhibitor of thrombin-induced fibrinogen polymerization. Further clotting studies in human plasma showed that due to the presence of prothrombin, an alternative target for aptamers, the antithrombin activity of aptamers in plasma samples may be significantly underestimated.

## Graphical abstract

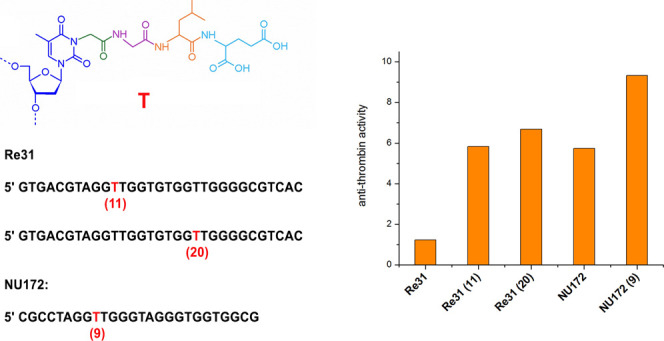

## 1 Introduction

Aptamers are single-stranded nucleic acids with a unique spatial organization that allows them to bind specifically and with high affinity to target proteins or small molecules. In this regard, aptamers are synthetic, cheaper analogues of antibodies. Due to the ease of chemical modification and conjugation, aptamers hold great promise for use in therapeutics, molecular diagnostics, and bioimaging.^1^ Aptamers are developed using the SELEX procedure,^2^ which is essentially a directed molecular evolution process. However, SELEX results can vary significantly in terms of binding affinity and target specificity of evolved aptamers. The low chemical diversity of natural nucleic acids partially explains the limited success rate in developing high-performance aptamers. One approach to solving this problem is the use of modified nucleic acid analogues in the selection process.^3,4^ Another approach to increasing binding affinity and target specificity is based on post-SELEX optimization. It primarily involves different types of chemical modification applied to specific nucleotides in the aptamer. Our recent efforts in this field have focused on a new optimization strategy that leverages on expanding the recognition interface of an aptamer.^5–8^ Although additional recognition subunit may be composed of arbitrary chemical groups,^5^ short peptide fragments have an advantage of high diversity due to the large selection of available amino acids.^6,7,9^ Depending on the surface context of the target protein, a suitable complementary peptide can be designed to modify an aptamer at a specific position. We recently reported on a series of tripeptide units that can form an additional recognition interface upon conjugation at a specific position of the 15-nucleotide thrombin-binding aptamer (TBA) or its analogues.^6–8^ The lead tripeptide, GLE, was able to significantly improve anticoagulant characteristics of TBA due to interactions with specific amino-acid residues of thrombin (Figure 1A–C). In practical aspect, the proposed strategy involves the design and screening of a small library of aptamer–peptide conjugates. While following certain structural considerations can be quite successful,^6,7^ the use of computational tools can greatly simplify this task. Thus, the BindCraft tool^10^ that generates peptide binders directly from a protein’s PDB structure has been examined recently with respect to selected protein targets.^11^

**Figure 1:**
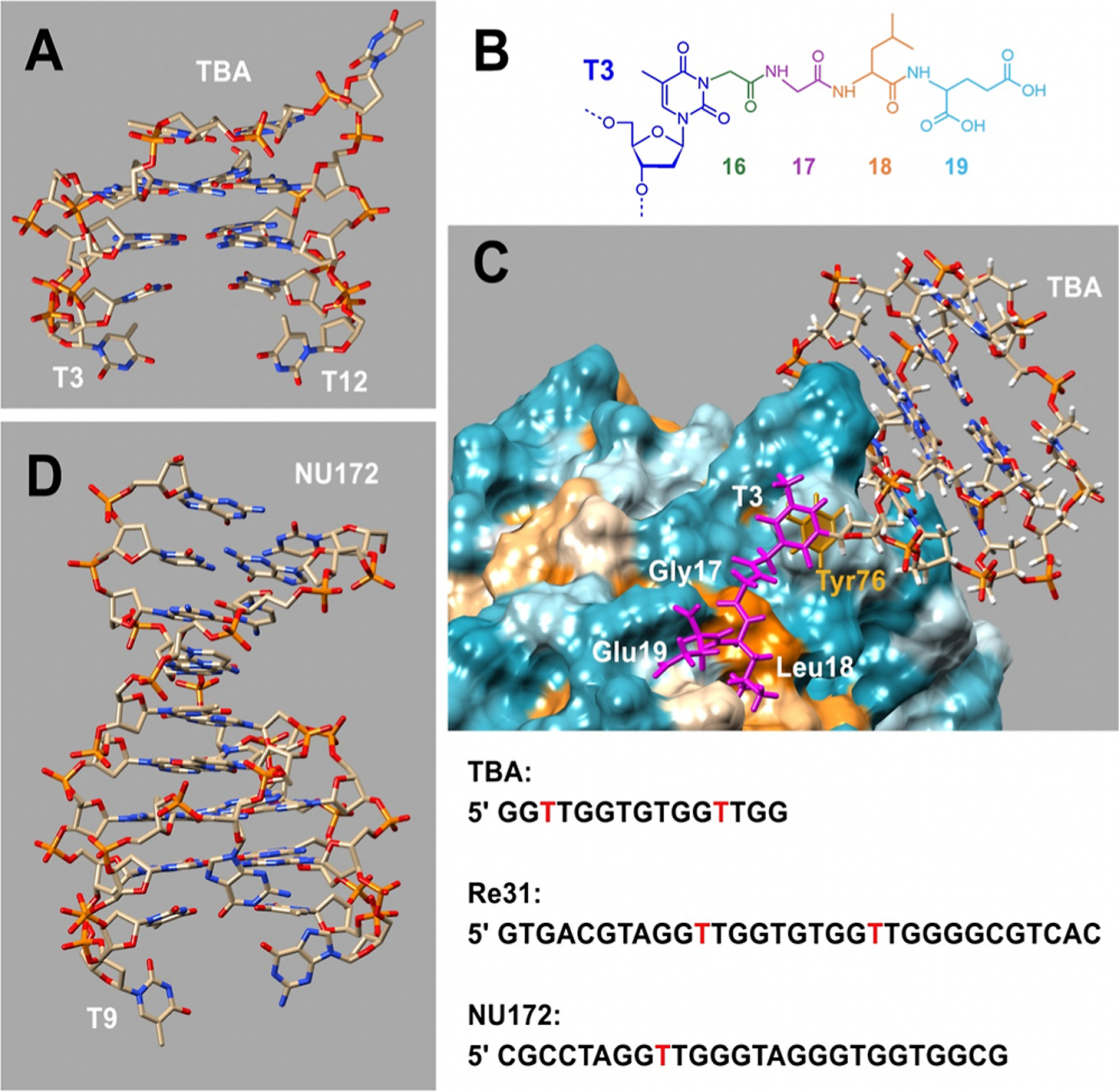
(A) Structure of TBA derived from its complex with thrombin (PDB 4DII). Due to pseudo C2 axis, either residue T3 or T12 can be modified without compromising aptamer’s ability to bind thrombin. (B) Chemical structure of the GLE subunit in the TBA–GLE conjugate. Carboxymethyl group 16 and Gly17 serve as a linker to residues 18 and 19, which interact with the amino acids of thrombin. (C) Snapshot of a complex of thrombin with the TBA–GLE conjugate (MD simulations). The protein surface is rendered by the hydrophobicity of the residues (blue-gold). Adapted under CC BY 4.0 license from Varizhuk et al. The Regioselective Conjugation of the 15-nt Thrombin Aptamer with an Optimized Tripeptide Sequence Greatly Increases the Anticoagulant Activity of the Aptamer. Pharmaceutics 2023, 15 (2), 604 (ref 7). Changes include modifying view angle, color scheme, and visibility of the K^+^ ion. (D) Structure of NU172 derived from its complex with thrombin (PDB 6EVV). The only position suitable for modification is T9 in a single TT loop. Sequences of aptamers TBA, Re31, and NU172 and positions of modification (shown in red) are given below panel (C).

Within the proposed approach, structural information about the organization of the aptamer–target complex has the highest priority, as suitable aptamer residues and the respective local surface context of the protein need to be identified. In this regard, TBA is a perfect model for the development of high-performance aptamer ligands with an extended recognition interface. The spatial structure of the 15-nt TBA itself and its complex with alpha-thrombin has been well established by various methods.^12–14^ NMR and crystallographic studies showed that TBA folds into a two-layer antiparallel G-quadruplex with two lateral TT loops and one central TGT loop (Figure 1A). Crystallographic studies of TBA–thrombin complexes revealed that the TT loops play a pivotal role in binding thrombin.^14^ Particularly, thymines T4 and T13 in the TT loops are involved in the interaction with residues Arg75 and Arg77A of thrombin exosite I. Thymines T3 and T12 are involved in hydrophobic interactions with thrombin residues (Ile79, Tyr117, and Tyr76).^14^ It is worth mentioning that due to the pseudo C2 axis, TBA can bind thrombin by two possible modes, as observed in the PDB structures 1HAO and 4DII.^13,14^ Depending on the binding mode, either T3 or T12 allocates in the proximity of Tyr76 of thrombin. It has been shown in previous studies that this region at the protein–aptamer interface allows for a wide variety of modifications at T3 or T12 without compromising aptamer’s ability to bind thrombin.^5,15^ The T3 position in the aptamer proved to be better suited for modifications than the T12 position, as T3-modified TBA variants were characterized by lower *K*_d_ values.^5^ Accordingly, the previously reported high-performance TBA–peptide conjugates were derived from a T3-modified aptamer variant.^6–8^

Bimodular thrombin aptamers are a family of mixed duplex–quadruplex structures that have been developed as advanced versions of TBAs.^16^ While some of the bimodular structures that bind to thrombin exosite I (Re31 and 31TBA) have a G-quadruplex module identical to TBA, the others (NU172 and its analogues)^16^ differ markedly in their loop composition. The presence of a duplex module in the aptamer structures of Re31, 31TBA, and NU172 proved to be highly advantageous with regard to anticoagulant activity and affinity to the protein.^16–19^

In the current study, we examined whether the bimodular thrombin aptamers Re31 and NU172 can be further optimized by adding the GLE peptide residue to the aptamer positions equivalent to the T3/T12 residues in TBA. Here, we report the synthesis of the aptamer conjugates, their biophysical characteristics, affinity to thrombin, and inhibitory potential. Furthermore, the ability of the studied aptamers and their peptide conjugates to bind prothrombin is discussed in the context of clotting studies with human plasma.

## 2 Results and Discussion

### 2.1 Design, Synthesis, and Biophysical Characterization of Re11, Re20, and NU9

The notably improved characteristics of the TBA–GLE conjugate are due to the specific interaction between the GLE tripeptide module and thrombin amino acid residues (Figure 1B,C). It was shown by MD studies that the Leu18 residue in GLE is allocated in the hydrophobic pocket of thrombin in proximity to the protein amino acids Leu65, Ile82, and Met84.^6,7^ The terminal residue Glu19 of the tripeptide formed electrostatic interactions with the thrombin amino acids Lys109 and Lys110. With regard to bimodular thrombin aptamers, the outcome of adding the GLE module would depend strongly on the similarity of different aptamer–protein complexes in the proximity of the thrombin residue Tyr76. A previous crystallographic study of the aptamer Re31 indicated that the quadruplex subunit remains mostly unchanged compared to TBA.^20^ The structural homology between these two complexes clearly points to the residues T11 and T20 in Re31 as structural equivalents of T3 and T12 in TBA (Figure 1 and Supporting Information Figure S1). Unlike Re31 and TBA, the bimodular aptamer NU172 has different loop compositions in the G-quadruplex module, a feature that notably affects the aptamer–thrombin interface. Structural studies of the complex between NU172 and thrombin^21^ revealed that the G13 residue in the central loop formed two hydrogen bonds with G18 in the GT loop. The latter, in addition to T19 and T10, interacts with the Arg75 of thrombin, thus building an asymmetric nucleotide framework around exosite I. This arrangement sets the position of a single T9T10 loop in the proximity to Tyr76. In the context of the current study, structural analysis of the NU172–thrombin interface identifies T9 as the only position suitable for conjugation with the GLE tripeptide (Figure 1D).

The chemical synthesis of GLE conjugates of the aptamer Re31 at positions T11 and T20 (Re11 and Re20) and of the aptamer NU172 at position T9 (NU9) was performed using N3-modified thymidine phosphoramidite^6^ (Figure 2A). The *p*-nitrophenyl carboxymethyl group is relatively stable and can be used in an automated oligonucleotide synthesis for preparation of oligonucleotides with an activated carboxyl group for further conjugation with peptides carrying a single reactive amino group. Protected solid-support-bound oligonucleotides were reacted with an excess of GLE tripeptide in DMF in the presence of DIPEA for 18 h. Further cleavage from the solid support and removal of the protecting groups with concentrated aqueous ammonia afforded the crude oligonucleotide–peptide conjugates (Figure 2B). In the final step, the conjugates Re11, Re20, and NU9 were purified by a combination of reversed-phase HPLC and electrophoresis in denaturing polyacrylamide gel. The presence of the tripeptide unit in the conjugates was confirmed by MALDI mass spectrometry (Supporting Information Figure S2). Unmodified aptamers TBA, Re31, and NU172, Cy5-labeled TBA, and previously reported conjugate TBA–GLE (TBA3)^7^ were used as reference species in biophysical and/or coagulation studies.

**Figure 2:**
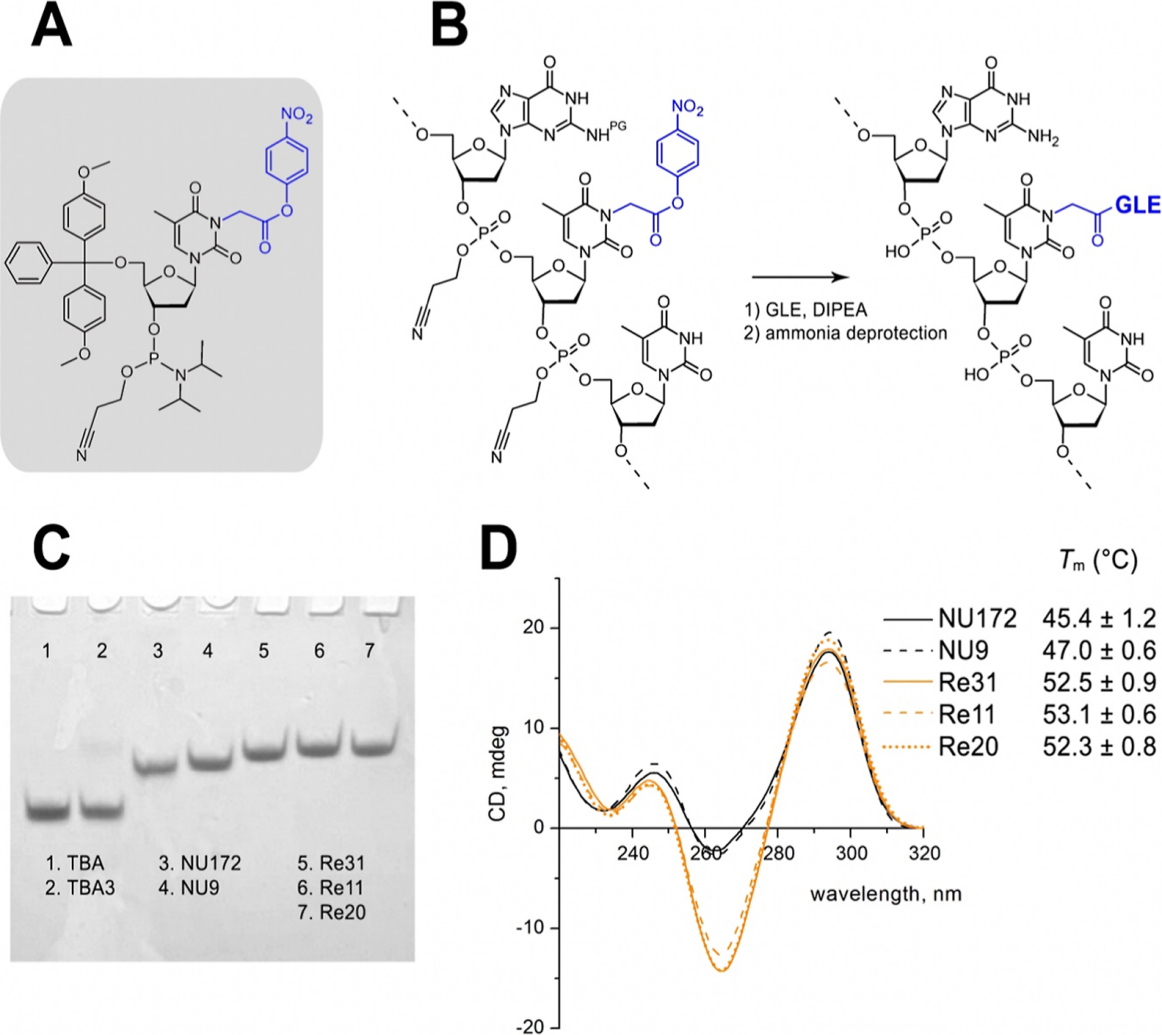
(A) Modified thymidine phosphoramidite with an activated carboxymethyl group. (B) Conjugation of the protected, support-bound modified oligonucleotide to the GLE tripeptide in the presence of DIPEA. Cleavage from the support and removal of the base protecting groups (PG) and cyanoethyl PG was performed using standard ammonia treatment. (C) Analysis of the aptamers TBA, NU172, and Re31 and their GLE conjugates by electrophoresis in denaturing polyacrylamide gel. (D) Circular dichroism (CD) spectra and *T*_m_ values for Re31, NU172, and their GLE conjugates in 100 mM K^+^ buffer.

The folding and unfolding of the quadruplex subunit in the aptamer conjugates Re11, Re20, and NU9 was monitored at 295 nm in a buffer containing 100 mM K^+^, revealing no significant difference between modified and unmodified aptamers (Figure 2C and Supporting Information Figure S3). Variations of *T*_m_ values between Re31 and the two tripeptide conjugates were within 1 °C, while the same value was approximately 1.5 °C for the pair NU172/NU9. This observation is consistent with the results of our previous study on the TBA3 conjugate.^7^ Earlier,^6^ we have shown that tripeptide side chains containing aromatic amino acids notably affect the thermal stability of the conjugates due to hydrophobic interaction with the T3 base. The GLE tripeptide is not engaged into intramolecular interactions with the modified pyrimidine base; therefore, it does not affect the *T*_m_ value. The melting and annealing curves of all Re and NU series aptamers at 295 nm showed insignificant hysteresis at a heating/cooling rate of 0.5 °C/min (Supporting Information Figure S3). The melting and annealing profiles at 260 nm, reflecting duplex module transitions, were also virtually identical in both aptamer series, regardless of the presence of the GLE module. The minimal effect of the GLE tripeptide on the aptamer structures was further confirmed by the CD spectra of the conjugates in 100 mM K^+^ buffer. Within each series of conjugates, the CD patterns remained nearly unchanged, showing positive peaks around 295 and 245 nm and a negative peak at 263 nm (Figure 2D).

### 2.2 Anticoagulant Activity of Re11, Re20, and NU9 in Fibrinogen Clotting Studies

In a series of our previous studies on TBA–tripeptide conjugates, we identified tripeptide modules that enhanced the anticoagulant characteristics of TBA. The lead tripeptide, GLE, was able to improve the activity of the aptamer approximately 6-fold upon conjugation at the loop thymine residue T3.^7^ To examine the effect of the added GLE module in bimodular aptamers under comparable conditions, we studied polymerization of fibrinogen in the presence of thrombin and aptamers using the same protocol. Polymerization was monitored by the increase in the absorbance of samples at 360 nm due to light scattering. Polymerization was carried out at 25 °C in phosphate-buffered saline (PBS) buffer at an aptamer concentration of 30 nM. The clotting time was estimated as the *t*_1/2_ value, i.e., the time required to reach 50% of the absorbance maximum. The results of the measurements are shown in Figure 3A,B as clotting curves and as a relative activity graph. Adding the GLE side chain to Re31 caused a remarkable shift of the clotting curve for both Re11 and Re20. Somewhat surprising, the conjugate Re11, which is a structural analogue of TBA3, appeared to be a slightly less potent inhibitor than Re20. This observation implies that the preferred selection of T3 over T12 for modification is justified only in the case of TBA. The GLE conjugates of TBA and Re31 expectedly showed clotting profiles in close proximity to each other, since the difference between the profiles of the unmodified aptamers was also insignificant. Unmodified NU172 demonstrated anticoagulant activity very similar to that of Re11, Re20, and TBA3. The NU9 conjugate proved to be the most potent thrombin inhibitor, demonstrating anticoagulant activity 9-fold greater than that of TBA. The relative anticoagulant activity defined as the *t*_1/2_/*t*_1/2_^TBA^ ratio is shown in Figure 3B. TBA3 and NU172 showed very similar activity, which is consistent with the results reported previously. The conjugate NU9 was approximately 1.6 times more potent than its unmodified precursor. The conjugates Re11 and Re20 showed a 4.8- and 5.5-fold increase in anticoagulant activity, respectively, compared to the parent aptamer Re31.

**Figure 3:**
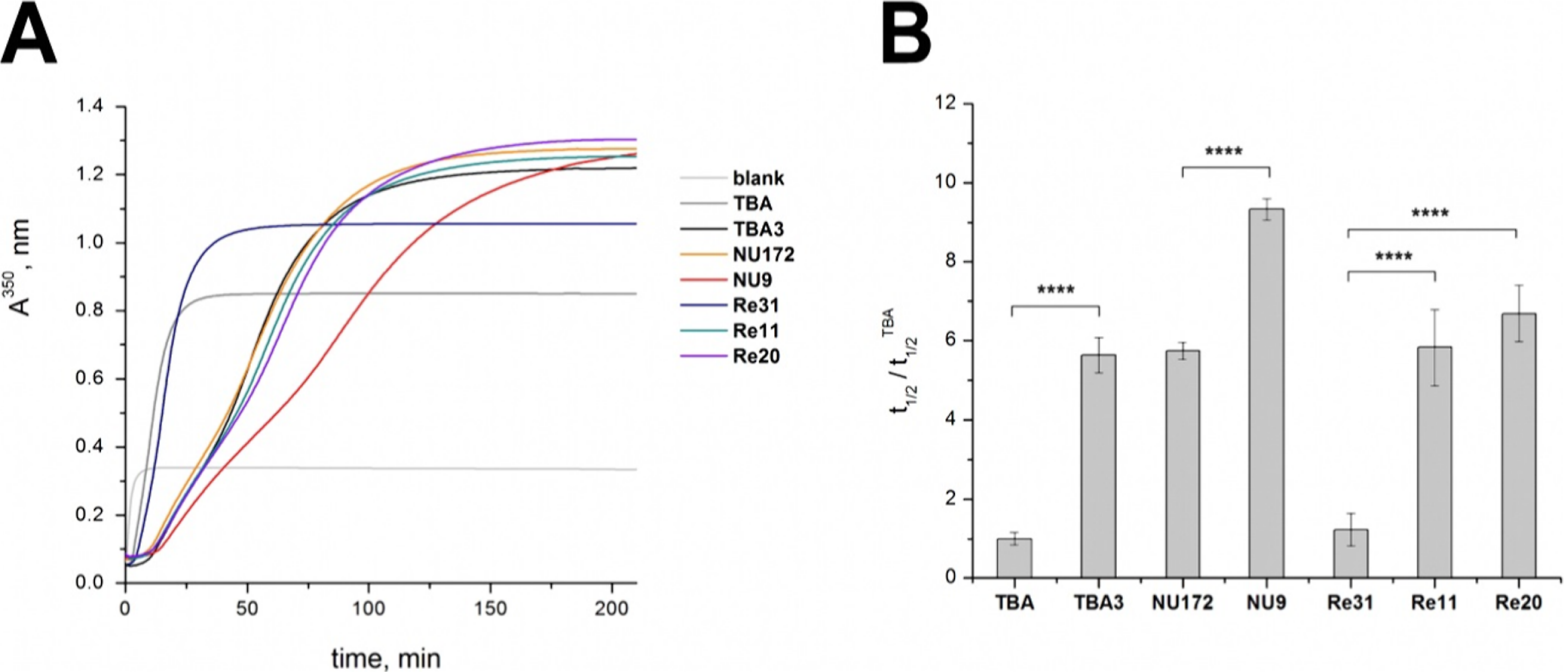
(A) Representative fibrinogen polymerization curves for aptamers and their GLE conjugates. NU9 was the most potent thrombin inhibitor. (B) Relative activities of aptamers defined as the *t*_1/2_/*t*_1/2_^TBA^ ratio. Shown are the mean values and standard deviations. The difference between each aptamer and its corresponding GLE conjugate is significant with *P* < 0.0001 (****).

### 2.3 Binding Affinity of Bimodular Aptamer Conjugates

Extending the recognition interface induces high anticoagulant activity of Re11, Re20, and NU9, which is assumed to originate from their enhanced binding affinity. In this study, we used microscale thermophoresis (MST) to determine the *K*_d_ values for the new aptamer conjugates. This method typically quantifies biomolecular interactions between a fluorescently labeled ligand and its target. In addition, MST can be applied for the affinity measurements in the case of competitive binding of two ligands to a target. This variant of MST suits well our purposes, as only one fluorescently labeled competitive aptamer is required. Furthermore, the use of unlabeled aptamer conjugates eliminates the potential effect of the fluorescent dye on the affinity characteristics. The mathematical model describing competitive binding of two different ligands to a protein molecule was developed previously.^22^ Cy5-labeled TBA (Cy5-TBA) was used as a competitive ligand. The *K*_d_ value for this aptamer (41.7 ± 8.4 nM) was determined independently by a standard MST protocol using 20 nM Cy5-TBA and thrombin concentrations in the range 0.12–10^3^ nM.

In competitive binding, a series of dilutions of the unlabeled aptamer is used at constant concentrations of Cy5-TBA and thrombin. Simulated curves of the bound and free fractions of Cy5-TBA in competitive binding with an arbitrary unlabeled aptamer (*K*_d_ = 10 nM) are given in Figure 4A. The simulations were performed for 100 nM thrombin and 20 nM Cy5-TBA, values that are within a reasonable concentration range. The effect of the increasing affinity of an unlabeled aptamer on the bound fraction curve is shown in Figure 4B. There is a good resolution between the curves in the *K*_d_ range of 1–10 nM. However, sub-nanomolar *K*_d_ values induce lower resolution under the chosen conditions and can therefore potentially be a source of larger errors. Furthermore, the significant difference between the *K*_d_ values for the labeled and unlabeled aptamer results in a noticeable deviation of the binding curve from the sigmoid shape. Experimental profiles of the thrombin-bound Cy5-TBA fraction in the presence of unlabeled bimodular aptamers and their GLE conjugates are shown in Figure 4C. Fitting procedure was carried out at fixed values for the concentrations of Cy5-TBA and thrombin (20 and 100 nM, respectively), and the *K*_d_ value for Cy5-TBA (41.7 nM). The affinity characteristics of bimodular aptamers and respective peptide conjugates perfectly matched their anticoagulant activities. The dissociation constant for Re11 (2.9 ± 1.4 nM) was very close to that of NU172 (3.1 ± 1.4 nM). Unmodified Re31 appeared to be the weakest binder in the studied series (10.1 ± 3.4 nM). Similar to clotting experiments, Re20 exhibited a higher affinity for thrombin (1.8 ± 1.1 nM) than Re11, thus confirming the prevalence of T20 over T11 as the preferred position for peptide conjugation. The most efficient binder, NU9, showed a *K*_d_ value of 0.24 ± 0.45 nM. Expectedly, the error increased in this case.

**Figure 4:**
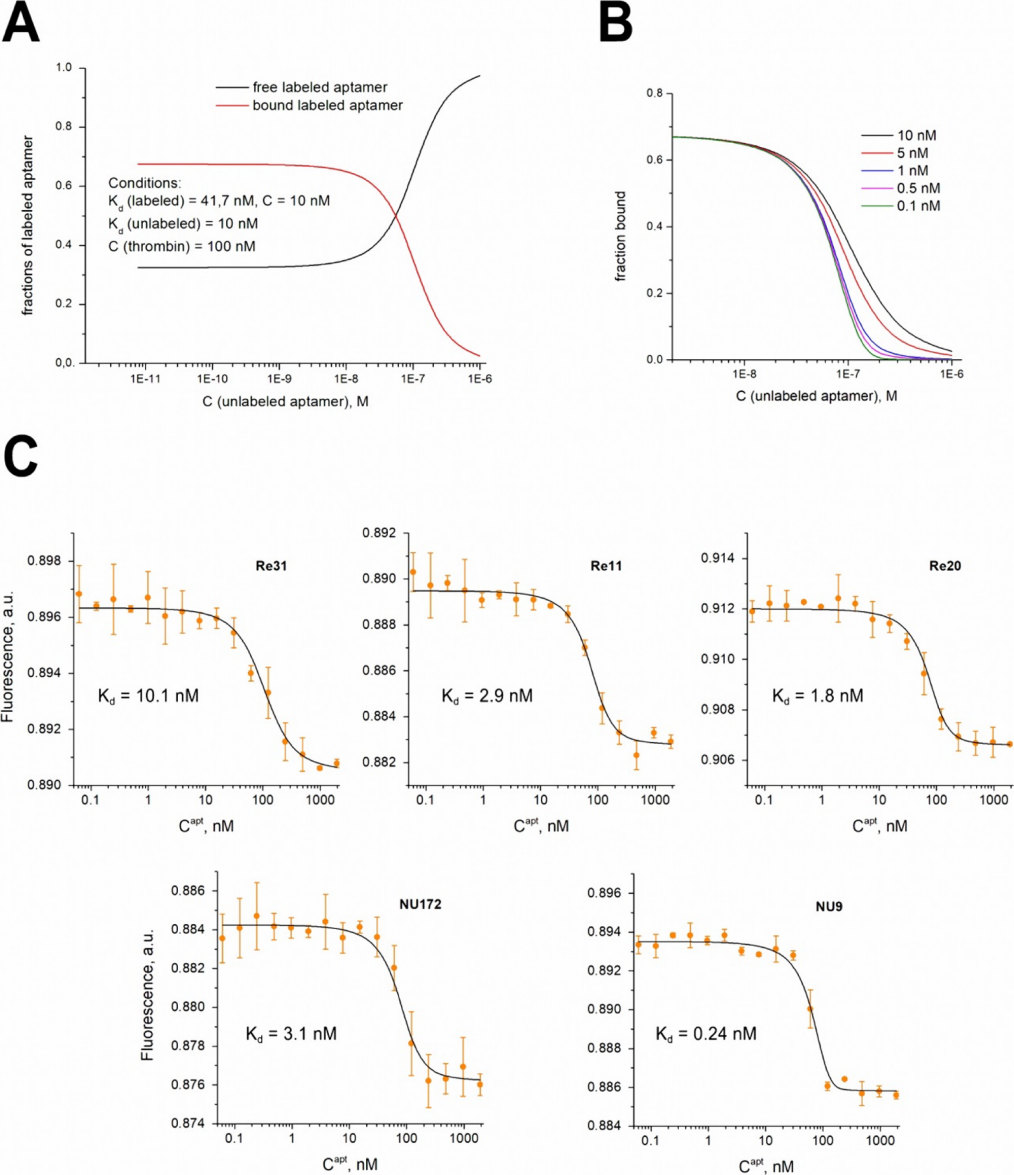
(A) Calculated fractions of free and bound Cy5-TBA in competitive binding to an arbitrary unlabeled aptamer (with assumed *K*_d_ = 10 nM) at 100 nM thrombin and 20 nM Cy5-TBA (*K*_d_ = 41.7 nM). (B) Effect of the increasing affinity of the unlabeled aptamer on the bound fraction of Cy5-TBA. (C) Experimental profiles of the thrombin-bound Cy5-TBA fraction vs the concentration of the unlabeled bimodular aptamer or aptamer conjugate. Experimental points are shown as mean values.

### 2.4 Clotting Studies in Human Plasma

We next studied the anticoagulant activity of bimodular thrombin aptamer conjugates in human blood plasma. Three standard blood hemostasis parameters—thrombin time (TT), activated partial thromboplastin time (aPTT), and prothrombin time (PT)—were measured for the conjugates and their parent aptamers using the automated hemostasis analyzer STA Compact Max (Diagnostica Stago S.A.S., Asnieres, France). The concentration of aptamers in the plasma samples was 0.8 μM, the value that allowed comparative measurements to be carried out within the analyzer’s detection limits (up to 220 s for TT). The results of measurements are given in Table 1. The effect of the added GLE peptide subunit was most prominent in the TT tests for Re11 and Re20, as well as for the TBA3 control. However, both NU172 and NU9, the highly potent thrombin inhibitors, showed only insignificant prolongation of TT. The two other parameters, aPTT and PT, which assess the intrinsic and extrinsic coagulation pathways, respectively, mostly reproduced the effect of the low activity of NU series aptamers, although on a smaller scale.

**Table 1:** TT, aPTT, and PT Values in Normal Human Plasma at an Aptamer Concentration of 0.8 μM (STA Compact Max)

sample	aPTT, s	PT, s	TT, s
blank^a^	31.4 ± 0.1^b^	12.3 ± 0.1	16.9 ± 0.1
TBA	42.3 ± 0.9	14.2 ± 0.2	55.0 ± 0.3
TBA3	38.7 ± 0.9	13.4 ± 0.1	94.4 ± 2.4
NU172	34.5 ± 1.8	13.2 ± 0.2	29.2 ± 0.3
NU9	32.3 ± 0.3	12.8 ± 0.3	19.0 ± 0.1
Re31	50.1 ± 0.2	14.5 ± 0.1	104.8 ± 2.2
Re11	42.0 ± 0.2	13.5 ± 0.1	120.4 ± 3.7
Re20	41.9 ± 0.6	13.8 ± 0.1	140.8 ± 6.3

a No aptamer added.

b Value ± SE (2 repeats).

The TT clotting test assesses the final step of the coagulation cascade that is triggered by the addition of exogenous thrombin. The notably reduced ability of NU series aptamers to inhibit thrombin should be associated with the binding of NU172 and NU9 to other plasma components, of which prothrombin (factor II, FII) is the most evident target.^23–25^ Prothrombin is a thrombin predecessor, whose concentration in blood is in the range of 1–2 μM.^26^ Exosite I of alpha-thrombin, the target protein of all aptamers used in this study, also exists in prothrombin and is referred to as proexosite I.^27^ Proexosite I has been shown to be a target for several DNA and RNA aptamers including TBA, Re31, and NU172.^23–25^ The aptamer binding to proexosite I has been proposed to interfere with the assembly of the catalytic prothrombinase complex of prothrombin with factors Xa and Va.^23^ A study on the interaction between prothrombin and TBA has shown a comparable affinity of TBA for thrombin and prothrombin.^23^ A more recent study has reported binding constants (K_b_) of the same order of magnitude for TBA, Re31, and NU172 upon prothrombin binding.^25^ Importantly, the affinity of the aptamers studied, including NU172, for prothrombin did not exceed the same for thrombin.

To confirm the effect of the competitive binding of plasma components with NU172, we verified whether preincubation of the aptamer with thrombin changes the results of TT measurements. For greater flexibility in the experimental design, further studies were carried out using a semiautomated two-channel coagulometer APG2-02 (EMKO, Moscow, Russia). When the NU172 aptamer at a concentration of 1 μM was preincubated with the thrombin reagent instead of plasma, the TT value notably increased. Preincubation of Nu172 in plasma resulted in a 1.3-fold increase in the TT value, while preincubation with thrombin increased the TT value by a factor of 4.5. These results confirmed that the binding of NU series aptamers to plasma components interfered with the inhibition of thrombin.

We then carried out plasma coagulation studies for a series of aptamers over a range of concentrations. In addition to normal human plasma, we used factor II-deficient human plasma (Instrumentation Laboratory, Bedford, MA, USA). The results of the clotting tests (TT) are shown in Figure 5. In normal plasma, we observed no significant activity of NU172 and NU9 up to a concentration of 1.5 μM. In contrast, most potent aptamers (Re11, Re20, and TBA3) exceeded the cutoff limit of measurements (set at 300 s) at concentrations greater than 1 μM. In factor II-deficient human plasma, the activity of all studied aptamers was remarkably higher. The TT value measurements were only possible at notably reduced aptamer concentrations in this case. Obviously, this effect should be associated with the absence of the competitive aptamer binder prothrombin. In factor II-deficient plasma, the TT values for NU series aptamers increased dramatically, confirming their high affinity for thrombin (Figure 5C). For comparative purposes, data on the relative activity of aptamers from different studies are presented in Figure 5D. Interestingly, the order of aptamer activity changed in the absence of prothrombin. Re11, Re20, and NU series aptamers were notably more potent than TBA3. Variations in the relative activity of aptamers and their conjugates most likely related to differences in experimental setup, plasma source, and sample composition. Nevertheless, the major result of the plasma clotting studies suggests that, in contrast to TBA-like structures, the activity of NU family aptamers exhibits critical dependence on the presence of prothrombin.

**Figure 5:**
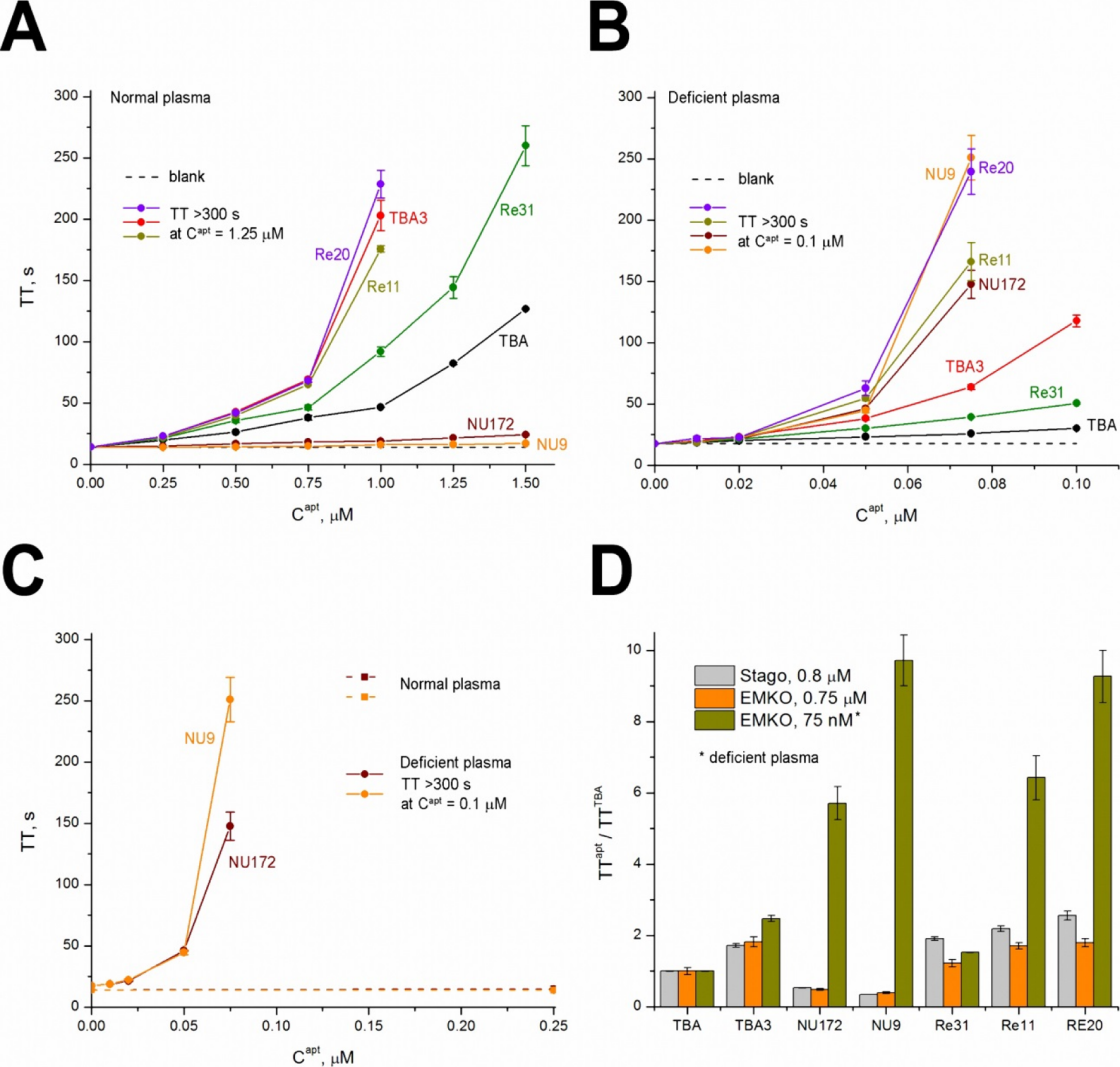
(A) TT values for aptamers and their conjugates in normal human plasma. Aptamer concentrations were in the range of 0.25–1.5 μM. (B) TT values for aptamers and their conjugates in FII-deficient human plasma. Aptamer concentrations were in the range of 0.01–0.1 μM. (C) TT values for NU172 and NU9 in normal and FII-deficient human plasma over a comparable concentration range. (D) Relative activity of aptamers and their conjugates from different experiments. Experimental points and bar heights are shown as mean values ± SE.

Since the affinity of a particular aptamer to prothrombin is expected to be of the same order of magnitude or lower than that for thrombin,^23,25^ the only plausible explanation for the observed effect consists in a specific combination of *K*_d_ values for thrombin and prothrombin under experimental conditions used. For a given *K*_d_ value of an aptamer for prothrombin (*K*_d_^PT^), equilibrium concentration of free thrombin in normal plasma can be roughly estimated using the Wang equation for competitive binding.^22^ For this estimation, the average plasma prothrombin concentration was assumed to be 1.4 μM (i.e., 0.7 μM after mixing with the TT reagent), and the thrombin concentration was calculated for an activity of 2500 IU/mg. In the calculations, we used the *K*_d_^T^ values for the thrombin–aptamer complexes derived from MST experiments.

The model graph of the unbound thrombin concentration vs the aptamer–prothrombin *K*_d_^PT^ values for NU172 and Re31 is shown in Supporting Information Figure S4. There is a range of reasonable *K*_d_^PT^ values for the two aptamers in the graph, when the concentration of unbound thrombin is higher in the NU172 sample. In this case, shorter TT values were expected. For example, *K*_d_^PT^ values of 3.5 nM for NU172 and 40 nM for Re31 would result in unbound thrombin concentrations of 4.2 nM and 2.1 nM, respectively. This analysis suggests that the presence of prothrombin in blood samples and different affinities of aptamers to thrombin, prothrombin, and prothrombin activation intermediates (prethrombins and meizothrombins)^28,29^ should be taken into account when studying the inhibitory characteristics of new anticoagulant aptamer derivatives.

## 3 Conclusion

Improving the affinity and inhibitory characteristics of aptamers through extending their recognition interface with a short peptide subunit is a post-SELEX strategy that proved to be successful for bimodular duplex–quadruplex aptamers Re31 and NU172, similar to previously reported GLE conjugates with TBA^6,7^ and non-natural TBA analogues.^8^ The structural similarities among TBA, Re31, and NU172 at the site of modification greatly facilitated the implementation of this strategy for bimodular aptamers. GLE conjugates of Re31 showed the highest gain in anticoagulant characteristics, while NU9 appeared to be the most potent thrombin inhibitor in the aptamer series studied. The affinity of bimodular aptamers and their conjugates for thrombin was in good agreement with the observed order of the antithrombotic activity. These results present scientific evidence for the potential of the new post-SELEX optimization strategy, which is based on modifying aptamers with short peptide side chains at the aptamer–protein interfaces. In addition, clotting studies with human plasma samples revealed an important aspect of activity measurements that is rarely discussed in the context of the anticoagulant activity of aptamers. Prothrombin, which is an alternative target for TBA, Re31, and NU172 family aptamers, is always present at relatively high concentrations in blood and plasma samples. Therefore, the concentration of active exogenous or endogenous thrombin can vary depending on the experimental conditions and the affinity of a particular aptamer for thrombin and prothrombin. Respectively, the results of activity measurement in plasma are expected to be underestimated to varying degrees compared with the results of model experiments. From a broader perspective, the dual activity of highly potent anticoagulant aptamers suggests the need for a more careful investigation of their interaction with prothrombin, as this interaction is apparently part of a complex exogenous regulatory mechanism of the blood coagulation cascade.

## 4 Materials and Methods

### 4.1 Materials and Instrumentation

Chemical reagents and solvents were purchased from various commercial suppliers and used without further purification. Standard reagents for automated oligonucleotide synthesis were purchased from Glen Research (Sterling, VA, USA). Modified thymidine phosphoramidite was prepared as previously described.^6^ The synthetic tripeptide GLE was purchased from Cloud-Clone Corp. (Wuhan, China). Research-grade thrombin from human plasma and reagents for the APG2-02 coagulometer were from Renam (Moscow, Russia). Reagents for the STA Compact Max Instrument were from STAGO Diagnostica (Asnières sur Seine, France). Human Factor II-deficient plasma HemosIL was from Instrumentation Laboratory (Bedford, MA, USA). Fibrinogen from human plasma was purchased from Sigma-Aldrich (St. Louis, MO, USA).

### 4.2 Automated Oligonucleotide Synthesis and Conjugation

DNA oligomers were synthesized using an ABI 3400 DNA/RNA synthesizer in DMTr-on mode. The coupling time for the modified thymidine phosphoramidite was set to 60 s. TBA–peptide conjugates were prepared by reacting the support-bound protected modified aptamer sequences with GLE tripeptide (10 mg) in a mixture of DMF (150 μL) and DIPEA (30 μL) for 18 h at 25 °C. Partial deprotection of the conjugates was carried out with concentrated ammonia for 6 h at 55 °C. DMTr-protected oligonucleotides were purified by reverse-phase HPLC (Hypersil ODS, 5 μm, 4.6 × 250 mm; 10–50% MeCN in 50 mM TEAA for 30 min). After removal of the 5′ DMTr group, oligomers were repeatedly purified by reverse-phase HPLC (0–25% MeCN in 50 mM TEAA for 30 min), with the control of the fractions by MALDI mass spectrometry and denaturing gel electrophoresis. Additional purification was carried out by preparative electrophoresis in denaturing 20% polyacrylamide gel (9:1) containing 7 M urea.

### 4.3 MALDI Mass Spectrometry

The mass spectra of TBA conjugates were acquired using a 4800 Plus mass spectrometer (AB Sciex, Framingham, MA, USA) in linear mode. 3-Hydroxypicolinic acid was used as a matrix. Before analysis, the samples were treated with Dowex 50WX8 in an ammonium form.

### 4.4 Ultraviolet Thermal Denaturation

The absorbance versus temperature profiles were obtained with a Cary 3500 UV–VIS spectrophotometer equipped with a Peltier cell holder (Agilent Technologies, Santa Clara, CA, USA). The melting experiments were performed at 295 and 260 nm in 10 mM potassium phosphate and 90 mM KCl (pH 7.5). The heating/cooling rate was 0.5 °C/min. The melting points were determined from derivative plots of the melting curves (295 nm). The oligonucleotide concentration was 2.5 μM. Before melting of each sample, a slow annealing (0.5 °C/min) was performed to allow the aptamers to fold.

### 4.5 Circular Dichroism Spectroscopy

CD measurements were performed at 20 °C with an aptamer concentration of 2.5 μM in 10 mM potassium phosphate and 90 mM KCl (pH 7.5) by using a Jasco-715 CD spectrometer (JASCO, Easton, MD, USA). The spectra were obtained at a bandwidth of 1 nm. Before measurement, each sample was slowly annealed (0.5 °C/min) from 90 to 20 °C.

### 4.6 Microscale Thermophoresis

The dissociation constants of the aptamer–thrombin complexes were measured with a Monolith NT.115 instrument (NanoTemper Technologies, Munich, Germany) using a Cy5 detection channel. In standard experiments, the concentration of the Cy5-labeled TBA aptamer was 20 nM in 10 mM Tris HCl, 100 mM potassium phosphate (pH 7.5), and 0.5% Tween 20. A standard series of dilutions in the same buffer was used for alpha-thrombin with the highest final concentration in a capillary being 1 μM. In competition experiments, the concentration of the Cy5-labeled TBA aptamer was 20 nM in 10 mM Tris HCl, 100 mM potassium phosphate (pH 7.5), and 0.5% Tween 20. The concentration of thrombin was 100 μM. A standard series of dilutions in the same buffer was used for the unlabeled aptamer with the highest final concentration in a capillary being 2 μM. Dissociation constants for unlabeled aptamers were calculated using DataFit Software (Oakdale Engineering, Oakdale, PA, USA).

### 4.7 Fibrinogen Clotting in the Presence of Aptamers

Polymerization was monitored by the increase in the absorbance using a Cary 3500 UV–VIS spectrophotometer (Agilent Technologies, Santa Clara, CA, USA). Human thrombin (50 μL, 10 U/mL) was added to a solution of fibrinogen (2 mg/mL) and aptamer (30 nM) in 1 mL of PBS in a quartz cuvette in a temperature-controlled cuvette holder of a spectrophotometer at 25 °C. Monitoring of the absorbance at 360 nm started immediately after the addition of thrombin and was stopped after the curve reached a plateau. The measurements were carried out in several experimental series. Each series included a blank sample (without aptamer) and TBA control. The clotting curve for each sample was measured at least in duplicate.

### 4.8 Plasma Clotting Assays (TT, aPTT, and PT) in the Presence of Aptamers

A plasma pool was prepared from blood samples derived from informed healthy donors. Blood was collected in 3.2% sodium citrate anticoagulant tubes and centrifuged for 12 min at 2200 rpm. The plasma was aliquoted and stored at −80 °C. TT, aPTT, and PT assays were performed with an automated hemostasis analyzer STA Compact Max (STAGO Diagnostica, Asnières sur Seine, France) using normal human plasma at a fixed concentration of aptamers. Stock solutions of the aptamers in PBS were added to the plasma samples to a concentration of 0.8 μM and incubated for 15 min at 25 °C before installation into the instrument. Standard protocols were used for the analyses. TT assays with normal or FII-deficient human plasma at different concentrations of aptamers were performed with a semiautomated two-channel coagulation analyzer APG2-02 (EMKO, Moscow, Russia). In a typical TT measurement, the aptamer was added to normal citrate human plasma or reconstituted human FII-deficient plasma (50 μL) and incubated at 37 °C for 3 min in a cuvette. Thrombin reagent (3 IU/mL, 50 μL) was added to a sample to initiate automatic measurement.

## Supplementary Material



## Notes

Supplemental figures and characterization data (PDF) (PDF)

## ⊥

Passed away on October 19, 2025.
