# Supplementary material for: High-Performance Thrombin Aptamers with a Peptide-Extended Recognition Interface
Source: ACS Omega. 2026 Jul 7;11(28):42946–55. doi: 10.1021/acsomega.6c05416 (PMC13393381; doi:10.1021/acsomega.6c05416)
Supplement: Supplementary file 1 [file ao6c05416_si_001.pdf]

Supplementary information

High performance thrombin aptamers with a peptide-extended recognition interface

Irina V. Varizhuk[1], Natalia A. Kolganova[1], Olga B. Gordeeva[2,3], Andrey A. Stomakhin†[1], Diana A. Talipova[1,4], Sergei A. Surzhikov[1], Edward N. Timofeev*[1]

[1]Engelhardt Institute of Molecular Biology, Russian Academy of Sciences, 119991 Moscow, Russia

[2]Petrovsky National Research Center of Surgery, 119991 Moscow, Russia

[3]Pirogov Russian National Research Medical University, 117997 Moscow, Russia

[4]Sechenov First Moscow State Medical University, 119991 Moscow, Russia

†Dr. Andrey A. Stomakhin passed away on October 19, 2025

Table of content

Page

Figure S1………………………………………………………………………….. 3

Figure S2………………………………………………………………………….. 5

Figure S3………………………………………………………………………….. 6

Figure S4………………………………………………………………………….. 7

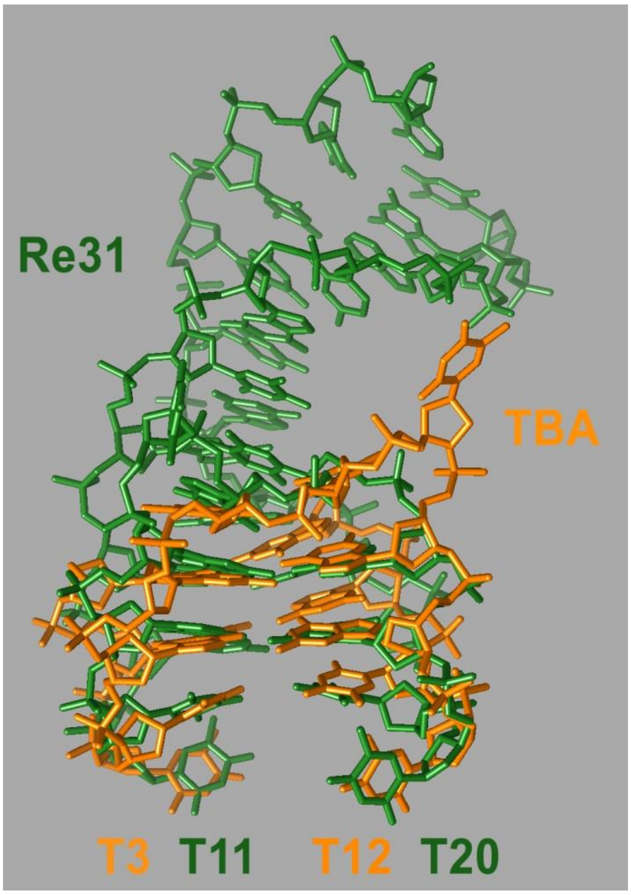

Figure S1. Structural homology between Re31 (green, PDB 5CMX) and TBA (orange, PDB 4DII) in their complexes with thrombin. T3/T11 and T12/T20 labels indicate homologous thymine residues in the TT-loops of Re31 and TBA.

50

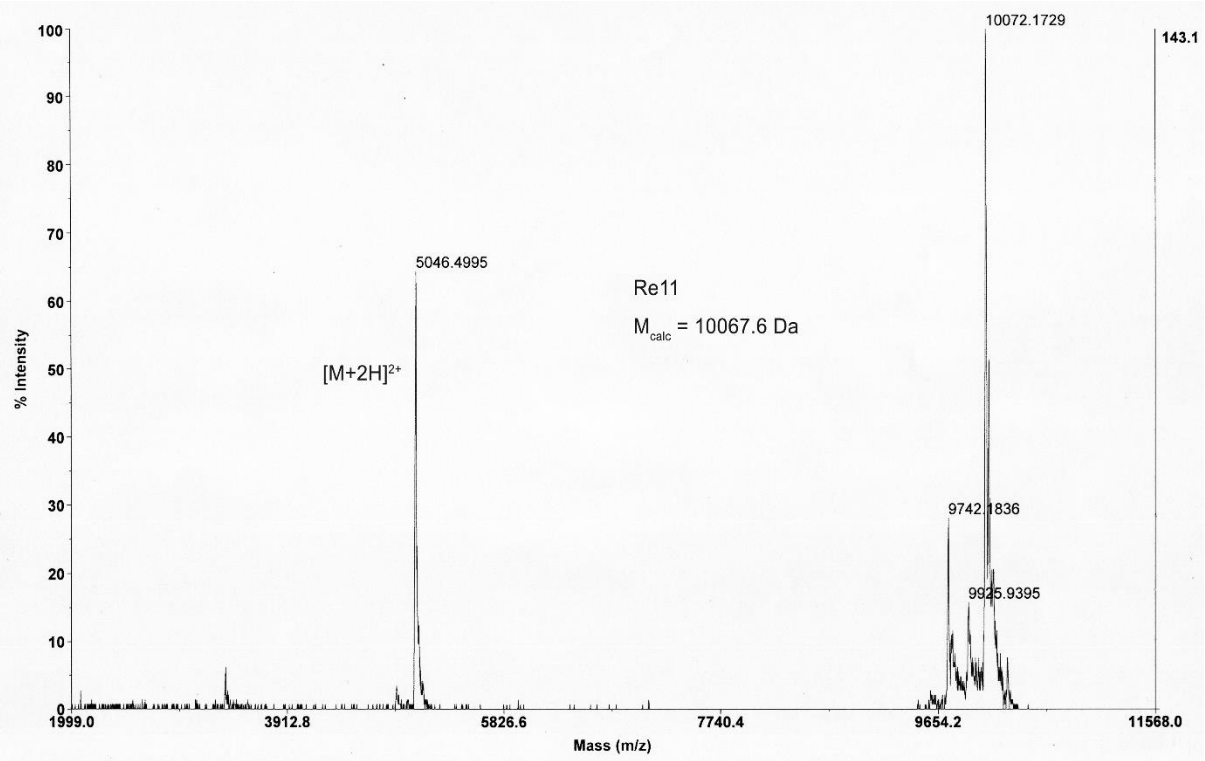
100
90
80
70
60
50
40
30
20
10
0
% Intensity
10072.1729
143.1
5046.4995
Re11
M_{calc} = 10067.6 Da
[M+2H]$^{2+}$
9742.1836
9925.9395
1999.0
3912.8
5826.6
7740.4
9654.2
11568.0
Mass (m/z)

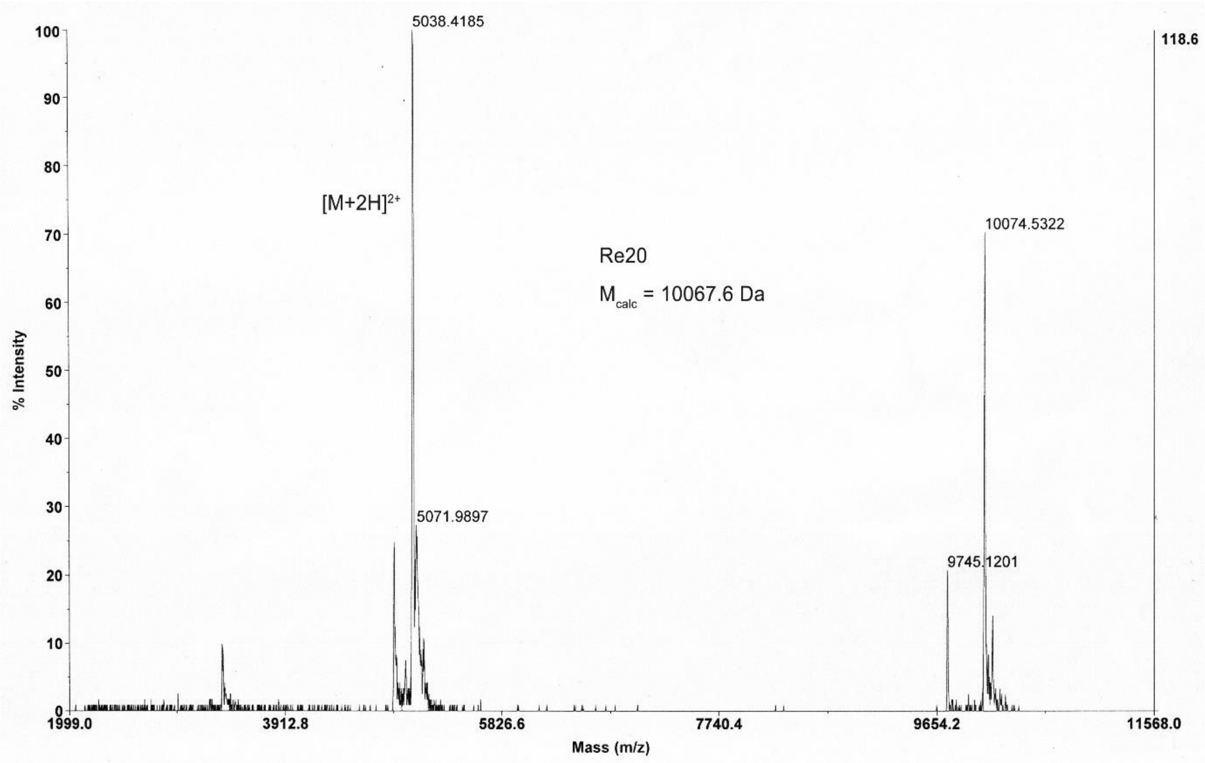
100
90
80
70
60
50
40
30
20
10
0
% Intensity
5038.4185
118.6
[M+2H]$^{2+}$
10074.5322
Re20
M_{calc} = 10067.6 Da
5071.9897
9745.1201
1999.0
3912.8
5826.6
7740.4
9654.2
11568.0
Mass (m/z)

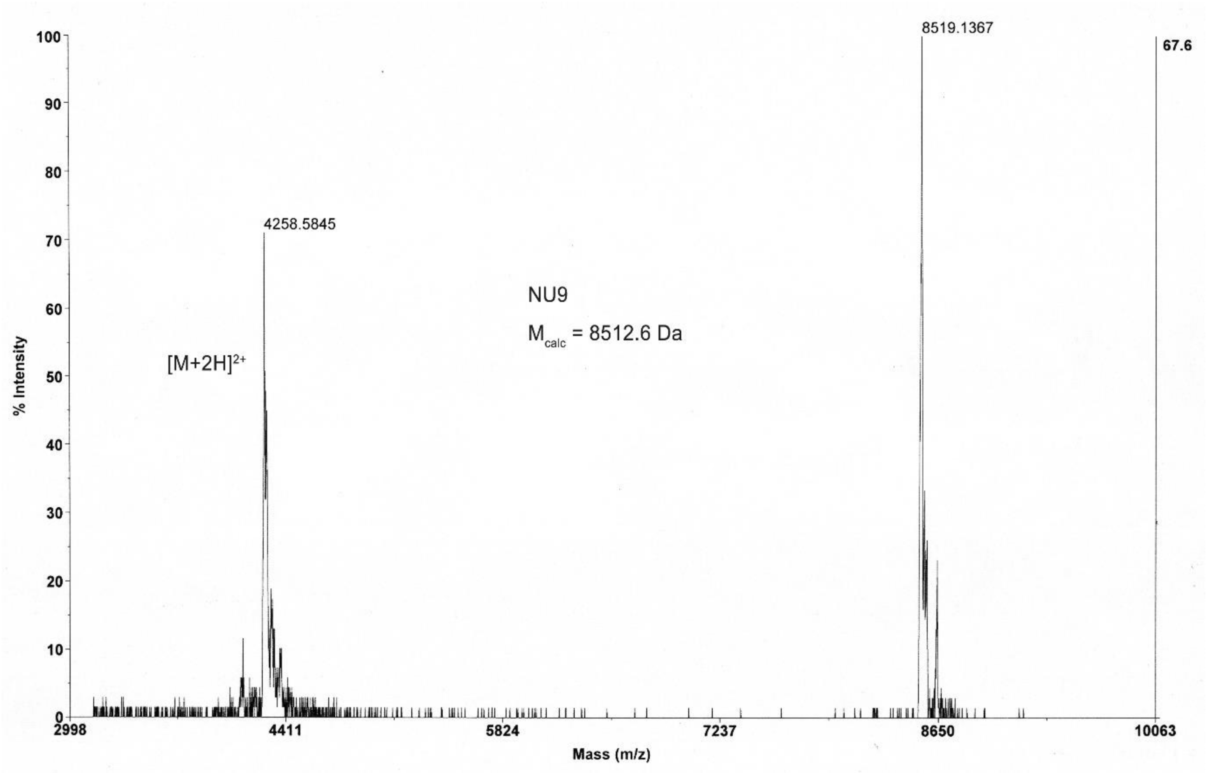

Figure S2. MALDI mass spectra of bimodular aptamer conjugates.

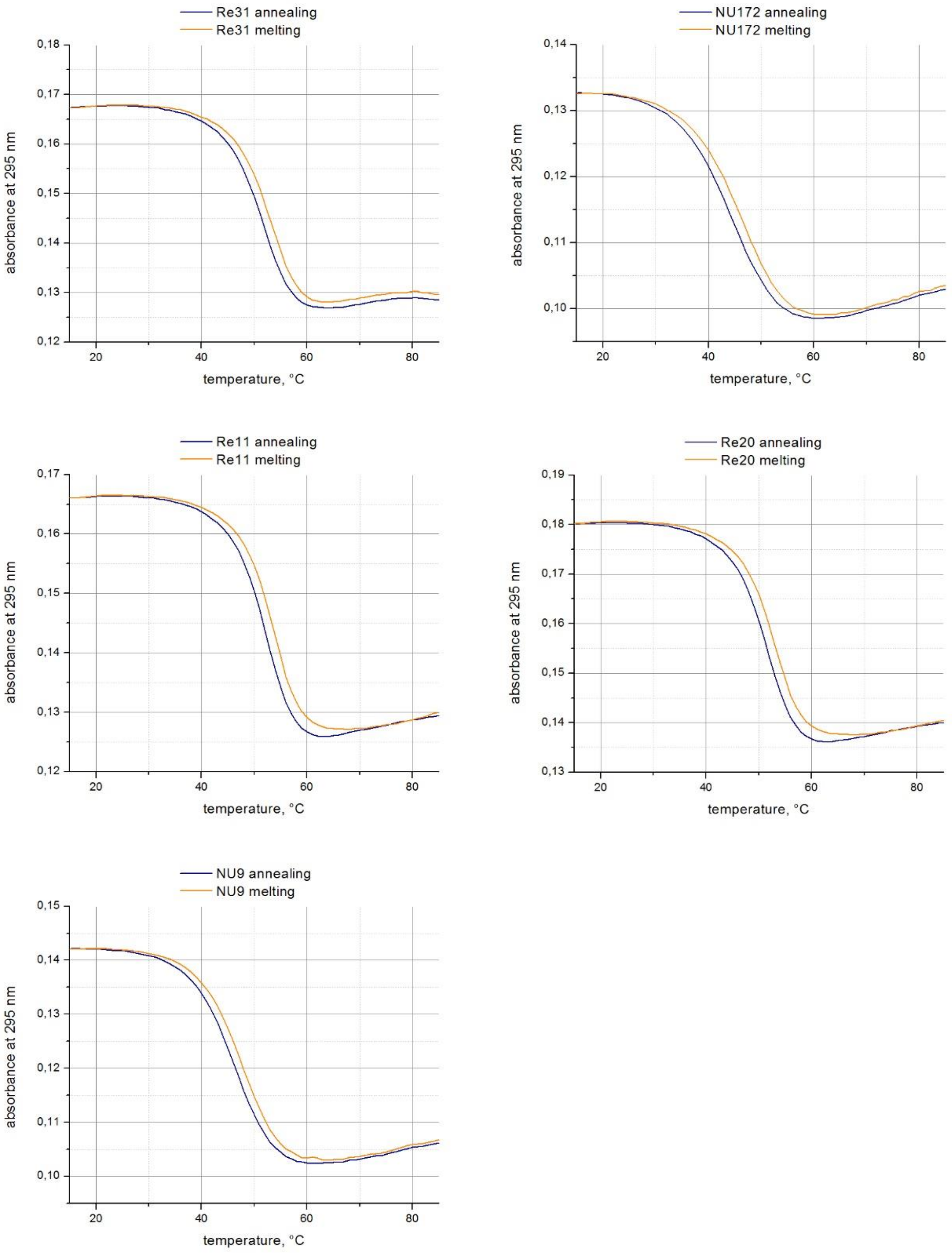

Figure S3. Melting and annealing profiles of Re31, NU172, and their GLE conjugates in 10 mM potassium phosphate and 90 mM KCl (pH 7.5). The heating/cooling rate was 0.5 °C/min.

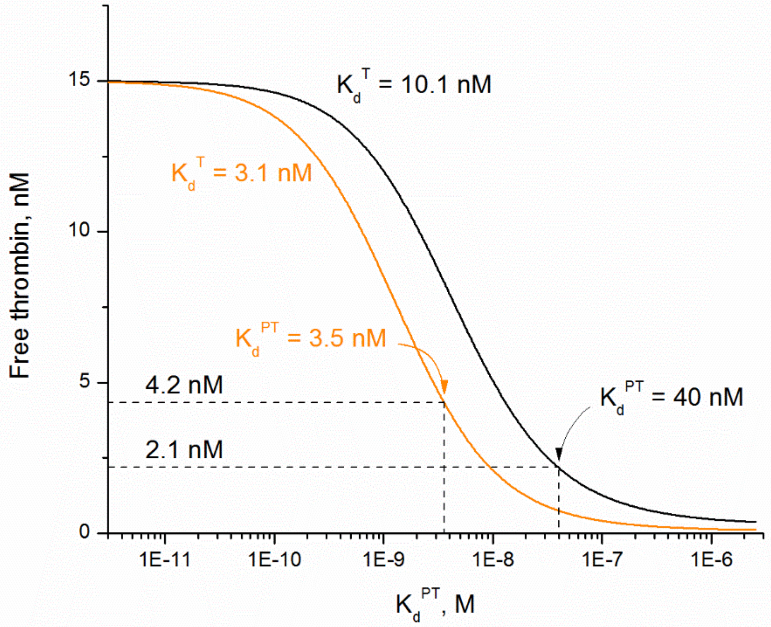

Figure S4. Model plot of the unbound thrombin concentration vs. the aptamer-prothrombin dissociation constant K_d^{PT} for NU172 (orange) and Re31 (black). The dissociation constant values (K_d^{PT}) shown are arbitrary to demonstrate the lower activity of the NU-series aptamers.
